# COVID-19: Changing Trends and Its Impact on Future of Dentistry

**DOI:** 10.1155/2020/8817424

**Published:** 2020-05-29

**Authors:** Parin Bhanushali, Farhin Katge, Shantanu Deshpande, Vamsi Krishna Chimata, Shilpa Shetty, Debapriya Pradhan

**Affiliations:** Department of Pediatric and Preventive Dentistry, TPCT's Terna Dental College, Navi Mumbai, India

## Abstract

The outbreak of coronavirus disease 2019 (COVID-19) rapidly escalated into a worldwide pandemic, creating a global health and economic crisis. It is a novel virus which is distinct from SARS-CoV and MERS-CoV, with Chinese horseshoe bats being the most probable origin. Transmission occurs primarily through droplet spread or contact routes. Due to the characteristics of dental settings, the risk of cross infection between dental health care personnel (DHCP) and patients can be very high. This article provides a brief overview of the structure of the virus, modes of transmission, and clinical features of COVID-19 disease. The aim of this article is to recommend infection control strategies and patient management protocols to provide optimum dental care and simultaneously prevent nosocomial infection in dental settings.

## 1. Introduction

Several epidemics (such as H1N1, H5N1, avian influenza, Ebola, SARS, Zika, and Nipah) have affected India and other countries in the past, which were successfully tackled with appropriate research [1]. The emergence of novel human pathogens and re-emergence of several diseases are of particular concern [2]. A novel human coronavirus initially referred to as the Wuhan coronavirus (CoV), currently designated as severe acute respiratory syndrome (SARS)-CoV-2, is responsible for the latest pandemic that is affecting human health and economy across the world [3]. On 30 January 2020, the WHO declared the Chinese outbreak of COVID-19 to be a Public Health Emergency of International Concern because of its rampant spread, thus posing a high risk to countries with vulnerable health systems. According to the WHO situation report (14 May 2020) update on COVID-19, there have been more than 42,48,389 reported cases and 2,94,046 deaths worldwide [4]. By imposing a nationwide lockdown, India has curtailed the spread of this virus to a certain extent; however, the total number of reported cases has crossed 78000 with approximately 2500 deaths and these numbers continue to rise [4].

Given the widespread transmission of SARS-CoV-2, healthcare providers are at an increased risk of contracting the infection and becoming potential carriers of the disease. According to Occupational Safety and Health Administration (OSHA), dental health care personnel (DHCP) are placed in very high exposure risk category as dentists work in close proximity to the patient's oral cavity [5]. Also, dental procedures involve the use of rotary instruments such as handpieces and scalers, which generate aerosols. Thus, a greater understanding of the structure of the virus, modes of transmission, clinical features, and testing methods is needed that can help to form protocols for dental practices to identify cases and prevent further spread of infection to the patients and DHCP.

## 2. Structure

SARS-CoV-2 is the seventh member of the family of coronaviruses that infect humans. Although similar to some betacoronaviruses, it is distinct from SARS-CoV and MERS-CoV. It is a novel virus belonging to the subgenus *sarbecovirus*, Orthocoronavirinae subfamily, with Chinese horseshoe bats (*Rhinolophus sinicus*) being the most probable origin [6]. It is an enveloped positive-stranded RNA virus with a diameter of 60–140 nm, spherical or elliptical in shape, and pleomorphic that shows a crown-like appearance under an electron microscope (*coronam* is the Latin term for crown) [7, 8].

## 3. Clinical Manifestations

Common symptoms at onset of illness include fever, nonproductive cough, myalgia, or fatigue; less common symptoms are sputum production, headache, haemoptysis, and diarrhoea [9]. Another common symptom is pneumonia which can be seen on chest X-ray or chest CT as multiple small patchy shadows and interstitial changes, remarkable in the lung periphery. Organ dysfunctions such as acute respiratory distress syndrome (ARDS), acute cardiac injury (shock and arrhythmia), acute kidney injury, and death can occur in severe cases [9, 10]. Age and comorbidity have been found to be risk factors for poor outcome [10].

### 3.1. Treatment/Management

Clinical management of COVID-19 is mainly symptomatic treatment. Severe cases require respiratory assistance with organ support in intensive care. No specific antiviral treatment exists, but antiviral, antimalarial, and biological drugs are administered in clinical trials [11].

## 4. Transmission

Although animal to human transmission was presumed to be the main mechanism, it has now been realised that infected patients, whether symptomatic or asymptomatic, are the main sources of transmission of the infection [10]. Current evidence suggests that person to person transmission occurs primarily via droplet spread or contact routes. Transmission via droplets occurs only in cases of close contact (within 1 metre) with those who have respiratory symptoms as there is a risk of oral/nasal mucosa or conjunctiva getting exposed to potentially exposing infected respiratory droplets when the person sneezes, coughs, or talks loudly [12].

In a dental setup, in addition to droplets, procedures on dental patients involving the use of high-speed handpiece or ultrasonic instruments may cause their secretions, saliva, or blood to aerosolize the virus into the surroundings. Thus, transmission can also occur through indirect contact by touching contaminated surfaces followed by self-delivery to the eyes, nose, or mouth [13]. The standard infection control measures which are otherwise followed in daily clinical work will thus not be effective enough to prevent the spread of COVID-19, especially when patients are in the incubation period, are unaware they are infected, or choose to conceal their infection [6].

## 5. Dental Setup

DHCP (dentists, dental hygienists, dental assistants, and receptionists) need to update their knowledge and skills regarding infection control and follow the protocols recommended by the relevant authorities to protect themselves and their patients against infections.

An attempt should be made to telephone triage all patients in need of dental care. Teledentistry can be of great assistance in the current pandemic situation. Newer technologies have not only enhanced the quality of management of dental patients but have also made possible their partial or complete management at distances of kilometres away from healthcare centers or dental clinics. The entire process of networking, sharing digital information, distant consultations, workup, and analysis is dealt with by a segment of the science of telemedicine concerned with dentistry known as “Teledentistry” [14, 15].

Based on the patients' signs and symptoms, a decision should be made to determine whether the patient needs to be seen in the dental clinic. Appropriate pharmaceuticals and detailed home care instructions should be provided by means of Teledentistry in situations where dental treatment can be delayed [5].

We have formed a table based on the information provided by American Dental Association (ADA) that helps to decide what constitutes a dental emergency; however, dentists should use their professional judgment in determining a patient's need for urgent or emergency care (Table 1) [16].

After a decision has been made that the patient needs to visit the dental clinic, the next step should be to evaluate the patients for signs and symptoms of COVID-19 infection to determine in which clinical setting they should be seen. According to Centers for Disease Control and Prevention (CDC) guidance, patients with active COVID-19 infection should not be seen in dental settings and should be referred for emergency care where appropriate transmission-based precautions are available [17].

A detailed history should be obtained from the patients by requesting them to fill the screening form for COVID-19 infection which should include the following questions: (1) Do you have fever or have experienced fever within the past 14 days? (2) Have you experienced a recent onset of respiratory problems, such as a cough or difficulty in breathing within the past 14 days? (3) Have you, within the past 14 days, travelled to countries with documented (SARS)-CoV-2 transmission? Or have you come into contact with people who have travelled to these countries? (4) Have you come into contact with a patient with confirmed (SARS)-CoV-2 infection within the past 14 days? (5) Are there people with documented experience of fever or respiratory problems within the last 14 days having close contact with you? (7) Have you recently participated in any gathering, meetings, or had close contact with many unacquainted people? [18].

**Figure 1:**
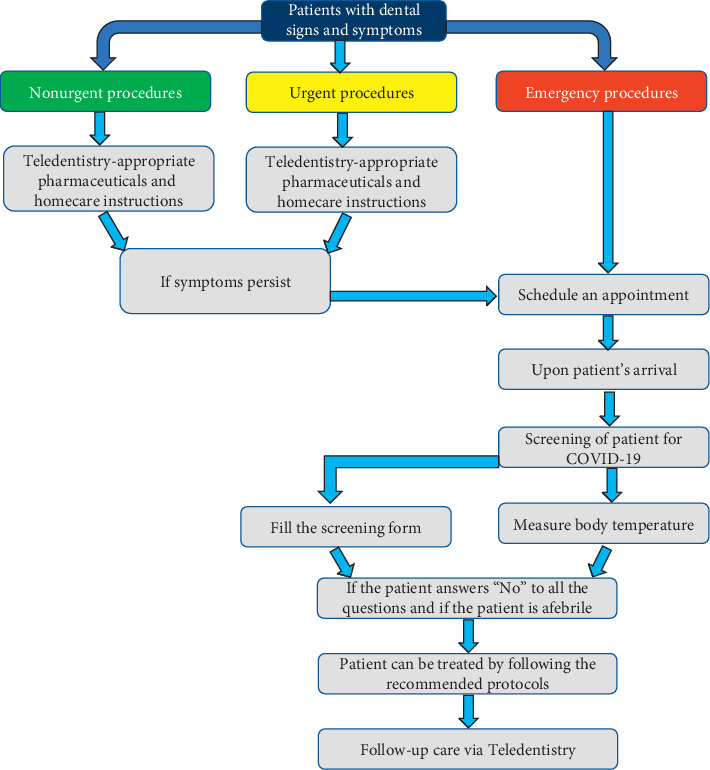
Management of dental problems during COVID-19 pandemic.

Upon patient's arrival, the body temperature of the patient should be measured using a contact-free forehead thermometer. If the patient answers “no” to all the questions and if the patient is afebrile, the patient can be treated by the dental surgeon following the recommended protocols (Figure 1). The ability to test patients who need dental care for SARS-CoV-2 is to be considered in order to restart dentistry in a sustainable way. Tests can be a strong tool to mitigate risks for patients and oral healthcare workers too [19].

## 6. Waiting Room

The Indian Dental Association recommends posting visual alert icons like signs and posters at the entrance and in strategic places to provide patients with instructions (in appropriate languages) about hand hygiene, respiratory hygiene, and cough etiquette. Instructions should include how to use tissues to cover nose and mouth when coughing or sneezing and to dispose of tissues and contaminated items in waste receptacles and how and when to perform hand hygiene [20].

Appointments should be scheduled such that social distancing can be maintained in the waiting room. Another alternative is for the patient to wait outside or in their vehicle and they can be contacted via telephone when it is their turn to be seen. It is recommended that the patients avoid bringing companions to their appointment, except for instances where the patient requires assistance. This can be communicated to the patient at the time of scheduling an appointment [17].

## 7. During Treatment

The National Task Force for Covid-19 constituted by Indian Council of Medical Research recommends the use of hydroxychloroquine for prophylaxis of SARS-Cov-2 infection for healthcare workers involved in the care of suspected or confirmed cases of COVID-19. The recommended dosage is 400 mg twice a day on day 1, followed by 400 mg once weekly for next seven weeks, to be taken with meals [21].

It is recommended that the highest level of personal protective equipment (PPE) available is used by dental surgeon and dental assistant while treating patients which includes gloves, gown, head cover, shoe cover, eye protection including goggles or a disposable/reusable face shield that covers the front and sides of the face, and a N954 or higher-level respirator. A combination of a surgical mask and a full-face shield can be used in situations when a respirator is not available [13].

Good hand hygiene is one of the best ways to prevent the spread of infectious diseases. A two-before and three-after hand hygiene should be followed in order to reinforce the compliance of hand washing. Specifically, the dental surgeon and the dental assistant should wash their hands before examining a patient, before performing any dental procedures, after touching the patient, after touching the surroundings and equipment without disinfection, and after touching the oral mucosa, blood, damaged skin, or wound [18].

Preprocedural mouth rinse with 0.5–1% hydrogen peroxide for its nonspecific virucidal activity against viruses or with 0.2% povidone-iodine is recommended as it might reduce the load of corona virus in saliva [22].

Intraoral X-ray examination is the most common radiographic technique in dental imaging; however, it can stimulate saliva secretion and coughing. Therefore, extraoral dental radiographies, such as panoramic radiography and cone beam computed tomography (CBCT), are appropriate alternatives during the outbreak of COVID-19 [6].

DHCP should avoid aerosol-generating procedures to the best and prioritize the use of hand instruments such as spoon excavators in combination with chemomechanical caries removal agents. However, if aerosol-generating procedure needs to be performed, it should be scheduled as the last appointment of the day [18]. Working from 10 or 11 o'clock position is recommended. In order to avoid splatter, eight o'clock position should be avoided. Use of rubber dam during such procedures is recommended as it could significantly reduce airborne particles in approximately three-foot diameter of the operational field by 70%. Four-handed dentistry with high volume suction for aerosols should be implemented along with regular suction [18]. Additional measures such as improving the quality of water, flushing of water from dental unit water lines, using antiretraction valves, antiretraction handpieces, and retrograde aspiration are strongly recommended to prevent cross infection [23].

## 8. Posttreatment

Because coronaviruses lose their viability significantly after 72 hours, many organizations have promoted a rotation and reuse strategy. It involves acquiring a set number of N95 masks (at least 5 as per the CDC), and rotate their use each day, allowing them to dry for long enough that the virus is no longer viable [24]. However, N95 respirators used during aerosol generating procedures or those contaminated with blood, respiratory or nasal secretions, or other bodily fluids from patients should be discarded [25].

Fumigation is not practical for dental operatory; however, measures such as mopping the floor with 1% sodium hypochlorite and disinfecting waterlines with 0.01% sodium hypochlorite can help reduce the risk of cross infection [26]. All biomedical waste pertaining to patient care should be carefully disposed from time to time through an authorized biomedical disposal agency [27].

Teledentistry as a form of Telehealth provides a pragmatic approach to assess and record the oral health status postoperatively and hence improve the overall delivery of oral care [28]. The dentist can monitor the treatment outcomes using mobile photography ensuring patient confidentiality and also provide educational videos regarding maintenance of oral hygiene for the benefit of the patient. With a paradigm shift in dental care practice in progress during the current pandemic situation, Teledentistry holds the prospects to attend the treatment needs of the patients without confrontation. It not only eliminates any chance of exposure to the virus but also decreases the service cost and helps in patient education and most importantly social distancing can be maintained. Teledentistry has changed the outlook of dentistry and never has it gained a stronger foothold in the practice as it probably holds during these times. So, it becomes imperative that the DHCP embrace this fundamental tool and apply it to its full potential.

## 9. Conclusion

Dental health care personnel need to understand the implications of potential transmission of the (SARS)-CoV-2 virus in a clinical setup. Hence, they need to keep themselves updated with any new information regarding this disease. New approaches such as Teledentistry will help dentists assist patients without adding the risk of cross infection. The recent state of affairs obligates the need to strike a balance between the safety of the healthcare professionals yet providing optimum dental care to the patients requiring emergency intervention.

## Figures and Tables

**Table 1:** What constitutes a dental emergency? (adapted from American Dental Association [15]).

Dental emergencies	Urgent dental care	Other urgent dental care
(i) Uncontrolled bleeding	(i) Severe dental pain from pulpal inflammation	(i) Extensive dental caries or defective restorations causing pain
(ii) Cellulitis or a diffuse soft tissue bacterial infection with intraoral or extraoral swelling that potentially compromises the patient's airway	(ii) Pericoronitis or third-molar pain	(ii) Manage with interim restorative techniques when possible (silver diamine fluoride, glass ionomers)
(iii) Trauma involving facial bones, potentially compromising the patient's airway	(iii) Surgical postoperative osteitis, dry socket dressing changes	(iii) Suture removal
	(iv) Abscess or localized bacterial infection resulting in localized pain and swelling	(iv) Denture adjustment on radiation/oncology patients
	(v) Tooth fracture resulting in pain or causing soft tissue trauma	(v) Denture adjustments or repairs when function impeded
	(vi) Dental trauma with avulsion/luxation	(vi) Replacing temporary filling on endo-access openings in patients experiencing pain
	(vii) Dental treatment required prior to critical medical procedures	(vii) Snipping or adjustment of an orthodontic wire or appliances piercing or ulcerating the oral mucosa
	(viii) Final crown/bridge cementation if the temporary restoration is lost, broken, or causes gingival irritation	
	(ix) Biopsy of abnormal tissue	
